# Clustering of antibiotic resistance of *E. coli *in couples: suggestion for a major role of conjugal transmission

**DOI:** 10.1186/1471-2334-6-119

**Published:** 2006-07-18

**Authors:** Susanne Lietzau, Elke Raum, Heike von Baum, Reinhard Marre, Hermann Brenner

**Affiliations:** 1 Department of Epidemiology, German Centre for Research on Ageing, Heidelberg, Germany; 2 Division of Clinical Epidemiology and Aging Research, German Cancer Research Center, Heidelberg, Germany; 3 Department of Medical Microbiology and Hygiene, University of Ulm, Ulm, Germany

## Abstract

**Background:**

Spread of antibiotic resistance in hospitals is a well-known problem, but studies investigating the importance of factors potentially related to the spread of resistant bacteria in outpatients are sparse.

**Methods:**

Stool samples were obtained from 206 healthy couples in a community setting in Southern Germany in 2002–2003. *E. coli *was cultured and minimal inhibition concentrations were tested. Prevalences of *E. coli *resistance to commonly prescribed antibiotics according to potential risk factors were ascertained.

**Results:**

Prevalences of ampicillin resistance were 15.7% and 19.4% for women and men, respectively. About ten percent and 15% of all isolates were resistant to cotrimoxazole and doxycycline, respectively. A partner carrying resistance was the main risk factor for being colonized with resistant *E. coli*. Odds ratios (95% CI) for ampicillin and cotrimoxazole resistance given carriage of resistant isolates by the partner were 6.9 (3.1–15.5) and 3.3 (1.5–18.0), respectively.

**Conclusion:**

Our data suggest that conjugal transmission may be more important for the spread of antibiotic resistance in the community setting than commonly suspected risk factors such as previous antibiotic intake or hospital contacts.

## Background

There is worldwide concern about the appearance and the rise of bacterial resistance to commonly used antibiotics [1,2]. Although the great majority of antibiotics are prescribed outside the hospitals and the human gut represents a large reservoir for potentially transferable antibiotic resistant *E. coli*, the potential role of transmission of antibiotic resistant bacteria between healthy individuals in the community is almost unexplored. We aimed to investigate the potential role of conjugal transmission of resistant *E. coli *compared to other suggested risk factors of carriage of resistant *E. coli*, like recent antibiotic intake or hospital stay, in a large community-based study.

## Methods

### Study population and data collection

This analysis was carried out as part of a large scale study primarily designed to investigate risk factors of carriage of antibiotic resistant *E. coli *in outpatient toddlers in Ulm, a city in Southern Germany. From July 2002 to July 2003 during the visit of a cooperating pediatrician, the accompanying parent and later on their spouses at home were also asked to participate in this study. Sociodemographic and medical data for the men (further referred to as "index persons") and their wives were obtained with a self-administered standardized questionnaire. In addition, all couples were asked to collect stool samples at home and to send them by mail to the study laboratory at the Department of Medical Microbiology and Hygiene at the University of Ulm (response rates among mothers and fathers 88.4% and 87.8%, respectively), where *E. coli *was cultured and resistance testing was carried out (see below). The study was approved by the ethical committees of the medical faculty of the University of Heidelberg, and of the chamber of physicians of the state of Baden-Württemberg. In this analysis, all couples were included, who gave written informed consent, and of whom both partners sent a stool sample from which *E. coli *could be cultured.

### *E. coli *culturing and antibiotic resistance testing

Stool samples were incubated on McConkey plates at 36 ± 1°C for 24 hours. Colonies of different phenotypes were tested with API20E (bioMérieux, France) in order to identify *E. coli *strains. Up to three phenotypically different *E. coli *colonies were kept frozen at -80°C in microbanks until susceptibility testing was performed. One of these colonies was randomly selected for resistance testing. Minimal inhibitory concentrations (MIC) were tested using micro well plates with various concentrations of antibiotics and an optical reader (Merlin, Germany) according to German national standards (DIN 58940) [3]. Weekly testing of *E. coli *strain ATCC 25922 was used for quality control. MICs for the following commonly employed antibiotics were determined (tested ranges and cutpoints of MIC in μg/ml are given in parentheses): ampicillin (1–128 μg/ml; >8), amoxicillin/clavulanic acid (1/2–128/2 μg/ml;>8/2), piperacillin/tazobactam (1/4–128/4 μg/ml;>32/4), cefpodoxime proxetil (0.25–32 μg/ml;>4), cefuroxime (1–128 μg/ml;>8), doxycycline (0.25–32 μg/ml;>4), gentamicin (0.25–32 μg/ml;>4), cotrimoxazole (2–28 μg/ml;>64), nalidixic acid (1–128 μg/ml;>32) and levofloxacin (0.0625–8 μg/ml;>2).

### Statistical analyses

We used descriptive statistics to characterize the index persons and their partners with respect to various sociodemographic and life-style factors, and to assess the prevalence of resistance of *E. coli *strains to the various antibiotics.

Next, potential determinants of antibiotic resistance among the index persons were evaluated. These analyses were restricted to ampicillin, doxycycline and cotrimoxazole as the resistance prevalences were too low for other substances to carry out meaningful analyses of associations. Apart from the presence of a spouse with an ampicillin, doxycycline or cotrimoxazole resistant *E. coli*, respectively, the following factors were considered as possible determinants of antibiotic resistance in bivariate as well as multivariable analysis (multiple logistic regression): antibiotic use within last three months, hospital stay within last 12 months, hospital/nursing home visit within last 12 months and frequent (daily) meat consumption. The time intervals were chosen to be as short as possible, but broad enough to include minimum number of subjects to allow for meaningful statistical analyses.

All analyses were carried out with the statistical software package SAS, Version 8.2.

## Results

In total, 206 couples were included in the analysis. Mean age of the index persons and their partners was 35 years (SD ± 5) and 32 years (SD ± 5), respectively. In total, 98% of the men were half- or full-time employed, whereas 72% of the spouses were housewives. Nine percent of the index persons and 12% of the partners had taken antibiotics within the last three months. A hospital stay within the last 12 months was reported by 6% and 26.6% of the index persons and their partners, and 58% and 62.1% had visited a hospital or nursing home during the last year, respectively. Sixty-one percent of the index person consumed meat daily, spouses reported daily meat consumption less often (51%).

The resistance prevalences of *E. coli *to various antibiotics were very similar among the men and their spouses. Among *E. coli *isolates cultured from stool of the index persons and their spouses, ampicillin resistance prevalences were 18.9% and 15.7%, respectively. Ten percent and 13.1% of the isolates from the index person and 9.2% and 14.9% of the isolates of their spouses carried cotrimoxazole and doxycycline resistance, respectively. Cephalosporine, gentamicin and quinolones resistance prevalences were three percent or less.

In bivariate analyses there were no indications that recent antibiotic intake, hospital stay or visit within the last 12 months or daily meat consumption were associated with antibiotic resistance (see table 1). Furthermore, no associations were found between antibiotic resistance and a variety of sociodemographic factors including the patients' age, education, occupation, number of family members in the household, square meter per person in the household, elderly persons in the household and a pet in the household (data not shown). By contrast, strong associations were found with antibiotic resistance among the partners. The associations for ampicillin resistant *E. coli *carried by both partners were highly significant. Resistance prevalences among the index persons were increased to 50.0%, 25.0% and 20.6% for ampicillin, cotrimoxazole and doxycycline, respectively, if the partner carried ampicillin, cotrimoxazole or doxycycline resistant *E. coli*, respectively, compared to resistance prevalences of 11.9%, 8.1% and 11.6%, respectively, observed otherwise.

**Table 1:** Bivariate analyses of the association of *E. coli *resistance to ampicillin, cotrimoxazole and doxycycline among the index persons.

		**Index person with ampicillin resistant *E. coli***				**Index person with cotrimoxazole resistant *E. coli***				**Index person with doxycycline resistant *E. coli***		
	**total**	**N**	**%**	**p-value**	**total**	**N**	**%**	**p-value**	**total**	**N**	**%**	**p-value**

Spouse with the same type of resistance												
No	168	20	11.9		186	15	8.1		172	20	11.6	
Yes	38	19	50.0	<.0001	20	5	25.0	0.02	34	7	20.6	0.2
Antibiotic use within last three months												
No	188	34	18.1		188	18	9.6		188	26	13.8	
Yes	18	5	27.8	0.3	18	2	11.1	0.8	18	1	5.6	0.3
Hospital stay within last 12 months												
No	194	37	19.1		194	20	10.3		194	26	13.4	
Yes	12	2	16.7	0.8	12	0	0	0.2	12	1	8.3	0.6
Visit of a hospital or nursing home within last 12 months												
No	86	20	23.3		86	9	10.5		86	11	12.8	
Yes	120	19	15.8	0.2	120	11	9.2	0.8	120	16	13.3	0.9
Meat consumption												
Sometimes/Never	81	19	23.5		81	11	13.6		81	12	14.8	
Daily	125	20	16.0	0.2	125	9	7.2	0.1	125	15	12.0	0.6

Table 2 shows the results of the multivariable analysis. After adjusting for antibiotic use within the last three months, a hospital stay during the last 12 months, visit of a hospital or nursing home within the last 12 months and frequency of meat consumption, spouses with ampicillin, doxycycline or cotrimoxazole resistant *E. coli *remained the main risk factor for the index persons to be colonized with resistant *E. coli*. In particular, carriage of ampicillin resistant *E. coli *by the partner was strongly and significantly associated with *E. coli *resistance to ampicillin in the index person (OR: 6.9; 95% CI: 3.1–15.5). Also carriage of cotrimoxazole resistant *E. coli *by the partner was associated with a significantly increased risk for the index partner to carry *E. coli *with cotrimoxazole resistance (OR: 3.5; 95% CI: 1.5–11.1).

**Table 2:** Multivariable analysis of potential risk factors of *E. coli *resistance to ampicillin, cotrimoxazole and doxycycline among index persons and their spouse.

	**Ampicillin resistance**		Cotrimoxazole resistance*		**Doxycycline resistance**	
	**OR**	**(95% CI)**	**OR**	**(95% CI)**	**OR**	**(95% CI)**

Spouse with the same type of resistance	6.9	(3.1–15.5)	3.5	(1.5–11.1)	2.0	(0.8–5.2)
Antibiotic use within last 3 months	1.3	(0.4–4.5)	1.2	(0.2–5.7)	0.4	(0.05–2.9)
Hospital stay within last 12 months	1.1	(0.2–6.1)	-	-	0.6	(0.07–5.1)
Visit of a hospital or nursing home within last 12 months	0.7	(0.3–1.5)	1.0	(0.4–2.6)	1.1	(0.5–2.6)
Daily meat consumption	0.8	(0.4–1.8)	0.5	(0.2–1.4)	0.7	(0.3–1.7)

*Hospital stay within the last 12 months was not included in the multivariable model of cotrimoxazole resistance, because there were no patients with recent hospital admission and cotrimoxazole resistant *E. coli*

## Discussion

The *E. coli *resistance prevalences in this community-based sample of couples from Southern Germany assessed in 2002–2003 were still on a relatively low level. The by far most important risk factor for the colonization with ampicillin, cotrimoxazole and doxycycline resistant *E. coli *was to live with a spouse carrying resistance to the respective antibiotic. Especially carriage of ampicillin resistant *E. coli *by the partner was significantly and strongly associated with ampicillin resistance in the index persons.

The resistance prevalences found in our study were similar to those found in a study conducted in the same region between May 1999 and January 2000 in Germany investigating *E. coli *resistance prevalences in the faeces of outpatients aged 40 years and older by the same methodology [4]. By contrast, much higher resistance prevalences in the community setting have been reported from studies conducted in the clinical sector in Germany, but direct comparisons are difficult due to selective sampling and different handling of the samples in the clinical setting.

Oral administration of antibiotics can influence the normal intestinal microflora and can lead to an overgrowth of resistant bacteria. But if the resistant bacteria persist for weeks or for months after the end of antibiotic treatment is discussed controversially [4-10]. Also hospital stay has previously been identified as potential risk factor for the carriage of resistant bacteria [11,12]. In our study, this factor did not play a major role. However, this finding may partly reflect lack of differentiation regarding important factors such as duration of stay, reason for admission or type of surgery. Furthermore meat consumption has been suggested as a potential sources of resistant bacteria and/or resistance genes [15], but in this study daily meat consumption was not associated with a higher risk for *E. coli *resistance. To our knowledge, our study was the first to address clustering of antibiotic resistant *E. coli *in a large sample of healthy couples from the community setting, and to assess the association of antibiotic resistant *E. coli *cultured from stool samples of the partner with own carriage of resistant *E. coli *strains.

Data on familial clustering of resistant bacteria in the outpatient setting are sparse. In a study from Sweden, stool samples were obtained from household members of outpatients with urinary tract infections [14]. In 16 (32%) samples of 51 family members trimethoprim resistance was found if trimethoprim resistant *E. coli *caused the urinary tract infection. Only one (2%) of 46 persons living in the same households carried trimethoprim resistant *E. coli *if the pathogen of the infection was sensitive to trimethoprim. Another study conducted in the United States in 1992 focused on the possible transmission of trimethoprim resistant *E. coli *from 33 day care center children to household members [15]. Family members of colonized children had significantly more resistant isolates than those from noncolonized children (26% vs. 2%). Although both studies included small numbers of families and were focused on trimethorprim resistance only and neither study explicitly addressed clustering between partners their findings are consistent with the suggestion strongly supported by our study that intrafamilial transmission is likely to play a major role in the spread of antibiotic resistance in the community setting.

Several limitations have to be kept in mind when interpreting our study results. The study was performed in 2002 to 2003 and therefore the resistance prevalences may not be as applicable to 2006. Furthermore the lack of associations of putative risk factors other than a partner with resistant *E. coli *must be interpreted with caution given the limited power of the study to address those associations. We relied on self-reports for our analyses regarding recent antibiotic intake, because not all antibiotics prescribed by the general practitioners are actually used and furthermore adults often receive antibiotic prescriptions from different physicians. Although self-reports of antibiotic intake may not always be reliable, major recall bias regarding antibiotic therapy within the last three months appears to be unlikely. However, our data and sample size limitations did not allow further stratification of previous antibiotic therapy by specific antibiotics, which may have diluted associations with resistance prevalences that might have been found for homologous substances.

In theory, clustering of antibiotic resistance in couples may reflect common sources of acquisition of resistant strains (or of resistant plasmids) as well as transmission of resistant strains (or resistant plasmids) between partners. Common sources of acquisition may be, for example, hospital visits or hospital stays of one partner (who might have frequent visits by the other partner), or common nutritional sources. Neither of these factors was found to play an important role in our study, however, which suggests that transmission is probably much more relevant in this sample.

Common sources of acquisition might also be other household members, such as children. Furthermore, potential transmission between partners may either be direct or indirect, e.g. via transmission from one partner to a child, who may then transmit resistant *E. coli *to the partner. Longitudinal studies will be needed for a definitive clarification of these potential alternative pathways, even though conjugal transmission appears to be the most plausible explanation for the observed patterns. However, the main finding of our study that couples are more frequently colonized with resistant *E. coli*, if their partner carries the same type of resistance, and that this factor is likely to be more important than other possible risk factors, e.g. recent antibiotic intake, holds regardless of the underlying mechanisms and pathway.

## Conclusion

The prevalences of antibiotic resistant *E. coli *in this population of young couples in Germany were not yet on a threatening level. Even though we only isolated *E. coli *from stool samples, it is to assume that first-line therapies for *E. coli *related diseases, like cotrimoxazole for urinary tract infection, should have been effective in most cases at the time of the study (2002–2003), taking into account that a large proportion of urinary tract infections are caused by self-infection [16]. Carriage of *E. coli *resistance by the partner is likely to be a key factor for carrying resistant *E. coli*, and this risk factor appears to be more important than other suggested potential risk factors among young adults in the community setting.

## Abbreviations

CI = confidence interval; *E. coli *= *Escherichia coli*; MIC = Minimal inhibitory concentrations; OR = Odds ratios; SD = Standard deviation.

## Competing interests

The author(s) declare that they have no competing interests.

## Authors' contributions

The study was designed by HB. SL coordinated the data collection along with the paediatricians. HVB supervised the microbiological testing. SL carried out the data analysis and drafted the manuscript, under the supervision of ER and HB. All co-authors were involved in the interpretation of the data and in the review of the manuscript. All authors read and approved the final version of the manuscript.

## Pre-publication history

The pre-publication history for this paper can be accessed here:


